# “Transparency” of Textbook Activities as Perceived by Chinese Middle School English Teachers With Different Professional Experience

**DOI:** 10.3389/fpsyg.2021.661014

**Published:** 2021-05-20

**Authors:** Hao Xu

**Affiliations:** National Research Centre for Foreign Language Education, Beijing Foreign Studies University, Beijing, China

**Keywords:** teaching materials, foreign language learning, foreign language teacher cognition, foreign language teacher education, classroom activities

## Abstract

Transparency of textbook activities, i.e., the degree of easiness for teachers to process and comprehend them, needs to be researched in terms of both materials *design* and materials *use*. This article reports on a study that investigates the impact of teachers’ professional experience and transparency of textbook activities (due to materials design) on teachers’ actual perception of the transparency of materials. The study adopted a mixed-methods design and collected quantitative data via a five-point scale survey from 115 secondary school teachers as well as interview data from 15 informants. Data analysis reveals that both teachers’ professional experience and transparency of textbook activities affect the degree of easiness teachers perceived as they understand and interpret the activities for pedagogical purposes. However, discrepancy exists between transparency in materials design and transparency in teachers’ perception. Lack of pedagogical knowledge about the relationship between teaching objectives, steps, and assessment and strong existing cognitive schemata developed from the stereotype of processing a familar set of teaching materials might hamper teachers’ perception of transparency.

## Introduction

How English as a Foreign Language (EFL) teachers use textbooks is of sustained interest to both teacher education and materials development researchers (Tomlinson, 2012). How teachers perceive and understand textbooks, classroom activities provided therein in particular, plays a pivotal role in teachers’ teaching practice (Tomlinson and Masuhara, 2012). Teachers’ understanding of textbook activities, for instance, that of the designed learning objectives and suggested learning process, informs their instructional design and classroom teaching (Ren and Han, 2016). Besides processing noticeable features of textbook activities that entail pedagogical instructions and suggestions, teachers also actively interpret textbook compiler’s intentions that underlie explicit descriptions of activity procedures (Bao, 2016). Therefore, it is important to more closely examine how teachers understand and interpret textbook activities, given the present paucity of research in this strand.

As part of teaching practice, understanding textbook activities involves a series of cognitive processes that are supported by teachers’ knowledge, skills, and beliefs, among many other psychological traits (Gao and Zhang, 2020). These traits are comprehensively represented in teachers’ experience, which, by and large, resides in their seniority in the teaching profession. Thus, experience should be investigated as a potential variable that impacts on how teachers understand textbook activities.

Presentation of textbook activities, or the way activities are shown in a textbook, also has impact on teachers’ understanding of them (Fuchs and Bock, 2018). A key property of such presentation is the transparency of activities, that is, the degree of easiness for teachers’ to process and comprehend activity procedures as well as pedagogical considerations that underpin the procedures (Xu, 2010). When teachers can easily understand their procedures and intentions, activities are considered transparent, while less transparent activities are more difficult to comprehend and interpret. Research shows that transparency of textbook activities is determined by comprehensibility of learning objectives, specificity of learning steps, and assessability of learning outcomes, all of which are manifested through presentation of textbook activities (Xu, 2021). Therefore, when activities are so presented as to allow teachers to easily identify intended learning objectives, sort out suggested learning steps, and implement classroom assessment, they will possess higher transparency.

Among the limited studies that examined how language teachers used materials or textbooks, researchers seemed to be primarily concerned about how teachers presented, in real classrooms, specific contents in the textbook (e.g., Sunderland et al., 2000) and how teachers taught consistently with or in a deviant manner from the intentions of textbook authors (e.g., Mohammaditabar et al., 2020). They, however, have rarely focused on teachers’ response to textbooks in cognitive terms, or more specifically, how teachers’ understanding of textbook activities is jointly influenced by teachers’ experience and transparency of textbook activities. As experience and transparency combined should constitute a more holistic picture, it is worth more research endeavors to reveal how these two variables contribute to teachers’ understanding of textbook activities in interactive ways. Therefore, the current study, as a pilot for a research project on a larger scale, explores the impact of the interface between experience and transparency. The research question this study aims to answer is: How is EFL teachers’ understanding of textbook activities influenced by teachers’ experience and transparency of the activities?

## Methods

### Research Design

This study adopted a mixed-methods approach to address the research question. In the quantitative phase, a 3 × 2 design was used to investigate the impacts of and interaction between the variables of experience (expert/experienced/novice) and transparency (high/low). Specifically, a sample of high-transparency activities and a sample of low-transparency activities, based on expert evaluation, were given the participants to evaluate their transparency using a five-point Likert scale. Then, some participants were interviewed about their thinking process as they evaluated the activities.

### Instruments

#### Questionnaire

As research shows that transparency of textbook activities is determined by comprehensibility of learning objectives, specificity of learning steps, and assessability of learning outcomes (Xu, 2021), the questionnaire survey of the current study focused on how comprehensible textbook activities are, as perceived by teachers, with regard to their teaching objectives, steps and assessment. The researcher first selected 10 samples of teaching materials for reading instruction, each including a narrative text as well as a complete set of pre-, while-, and post-reading activities (i.e., a warm-up activity to introduce the topic or theme to the learners, a series of activities to train learners to identify and understand information as well as pragmatic features of the text, and a series of activities to help learners internalize key vocabulary and grammar). All of the 10 samples were chosen from English textbooks for the 8th grade approved by China’s Ministry of Education but *not* used in Guangdong Province where all the participants were teaching. The 8th grade is the 2nd year in junior high school in China, commonly seen as a more representable year than the 1st year (featuring transition from primary school to secondary school) and the 3rd year (featuring heavy emphasis on preparation for the entrance examination to senior high school). Nine experts were invited to rate the 10 samples with scores (1–5) on the transparency of their activities in terms of teaching objectives, steps, and assessment; that is, each expert produced three corresponding scores for each of the samples, the mean score of which was then calculated. Three of the experts were university professors specialized in research on EFL teaching in schools, three were expert junior high school teachers, and three were senior editors from publishers that compiled EFL textbooks for schools. The samples with the highest and lowest mean scores were finally selected as sample materials to be used to elicit teacher participants’ evaluative responses.

Coupled with the selected samples of textbook activities, nine statements were produced as questionnaire survey items, each three pertaining to one of the three facets of teaching objectives, steps, and assessment. Examples of the items are shown below:

I can understand what students need to achieve after learning this lesson. (objectives)
I can figure out why the activities are arranged in such a sequence. (steps)
I can see which activity/activities should be used to conduct classroom assessment. (assessment)

#### Interview

The interview, which was implemented after the questionnaire survey, was so designed as to further examine how teachers approached the sample materials as they perceived their transparency. It also aimed to examine why teachers interpreted the sample materials as of high or low transparency. In short, the interview focused on how the sample materials were perceived as easy or difficult to understand with reference to their teaching objectives, steps, and assessment.

### Participants

Participants were 115 EFL teachers (19 males), 77 of whom were teaching the 8th grade and 38 of whom had just taught the 8th grade in the previous school year. They were from 26 junior high schools in Guangzhou and Shenzhen, two cities in Guangdong, which is a southern province in China. Among them, 32 teachers were expert teachers, with more than 20 years of teaching experience: they were either *teji* teachers, an official title granted to expert teachers by education authorities, or recipients of first-rank provincial or national teaching awards. The cohorts of experienced teachers and novice teachers were composed, respectively, of 49 teachers with 10–15 years of teaching experience and 34 teachers with 1–3 years of teaching experience, which followed the widely accepted criterion for recognizing teachers’ professional development stages (Farrell, 2012). All of the 115 participants signed written consents as they submitted their responses to the quantitative survey.

Fifteen of the participants, five from each of the three cohorts (expert/experienced/novice), were randomly selected to be interviewed, to which they also gave their formal consent as interview informants.

### Data Collection

The 115 teacher participants browsed the two sample materials for no longer than 40 min, which was sufficient time for the reading and analysis of both the texts and the activities. They then evaluated the transparency of the activities as they responded to a five-point Likert scale questionnaire that consisted of nine items of statements, each three pertaining to one of the three facets, which were teaching objectives, steps, and assessment. They responded to the same nine items twice, for each of the two sample materials, respectively. Participants read the nine statements via an online questionnaire survey service and entered a number (1–5) indicating how true they were to their own impressions of the sampled activities, from very untrue (1) to very true (5).

Five participants from each of the three cohorts (expert/experienced/novice) were randomly selected to be interviewed, totaling up to 15 interview informants. In individual interviews, each of the 15 participants was invited to further comment on the transparency of activities in the two sample materials, that is, how easy or difficult it was for them to understand and interpret the teaching objectives, steps, and assessment of the activities. However, the researcher persistently refrained from use or mention of the term “transparency” to prevent hinting at the research aim. In interviews, participants were also encouraged to recall and share their thinking process as they evaluated the two sample materials.

### Data Analysis

Analysis of quantitative data obtained from the materials evaluation survey began with reliability tests to confirm internal consistency across the three dimensions of the survey (objectives, steps, and assessment). The α values for high- and low-transparency materials samples were 0.683 (*N* = 115) and 0.630 (*N* = 115), respectively, both indicating acceptable internal consistency. Second, ANOVA (repeated measures) was conducted to calculate the main effects of and interactions between experience and transparency, the two independent variables. Third, two tests of ANOVA were conducted to more closely examine the impact of experience on evaluative scores given to high- and low-transparency materials, respectively, followed by a *post hoc* test to further determine relations between the three cohorts (expert/experienced/novice) if the *F* value was found significant.

Interview data were transcribed and proofread. It was then analyzed in line with the strategy of reduction–comparison–display (Miles et al., 2020). Data were first carefully read through as excerpts were identified and coded, which were closely related to research themes, thus effectively reducing the volume of data to be examined in further analysis. In the second round of analysis, comparison was made to reveal the difference in materials evaluation by teachers of different experience. The last round of analysis aimed at organizing the qualitative data for display as to corroborate and further elaborate results produced by quantitative data analysis. Interview data were analyzed in Chinese and translated into English when presented in this article.

## Results and Findings

### Textbook Evaluation Survey

Table 1 below is a summary of teacher participants’ evaluation scores of the transparency of the two sample materials, one designed and expert-evaluated as of high transparency, and the other of low transparency.

**TABLE 1:** Teachers’ evaluation scores on transparency of sample materials.

	**Number**	**High transparency**		**Low transparency**	
		***M***	***SD***	***M***	***SD***
Expert	32	3.88	0.292	2.19	0.292
Experienced	49	2.56	0.376	2.10	0.345
Novice	34	2.65	0.331	2.18	0.313
Total	115	2.95	0.671	2.15	0.322

As can be seen, teachers gave a higher mean score to activities in the high-transparency sample (*M* = 2.95) than to those in the low-transparency sample (*M* = 2.15), which concurred with expert evaluation in materials selection. Second, although teachers of different experience rated the low-transparency sample with similarly low scores, they seemed to be divided as to the transparency of the high-transparency sample: expert teachers rated it with a much higher score than experienced and novice teachers.

Repeated measures ANOVA was conducted to determine if the two independent variables—transparency of sample materials (high/low) and experience of teachers (expert/experienced/novice)—significantly affected the rating (see Table 2 above). Results showed that transparency and experience both significantly influenced the evaluation scores and that interaction between transparency and experience was also significant. LSD *post hoc* multiple comparisons among the three experience cohorts showed that expert teachers rated significantly higher than experienced teachers (*MD* = 0.706, *p* = 0.000), that expert teachers rated significantly higher than novice teachers (*MD* = 0.623, *p* = 0.000), and that experienced and novice teachers did not rate significantly higher or lower than each other (*p* = 0.109). In other words, results demonstrated that expert teachers rated significantly higher than experienced and novice teachers on the transparency of sample materials.

**TABLE 2:** ANOVA on effects of transparency and experience.

	***F***	***Sig.***
Transparency	376.602	0.000
Experience	98.498	0.000
Transparency × Experience	76.952	0.000

However, in the aforementioned multiple comparisons, high- and low-transparency samples were not treated separately in statistical analysis, so it was necessary to split them and to determine if expert teachers rated both of them significantly higher than others. ANOVA was thus conducted to examine the impact of experience (expert/experienced/novice) on the rating of the high-transparency sample and on that of the low-transparency sample, respectively. Results showed significant impact in the case of high-transparency sample (*F* = 164.371, *p* = 0.000) and no significant impact in the case of low-transparency sample (*F* = 1.030, *p* = 0.360). LSD *post hoc* multiple comparisons for the case of high-transparency sample showed that expert teachers rated significantly higher than experienced_1_ and novice_2_ teachers (*p*_1_ = 0.000, *p*_2_ = 0.000) and that experienced and novice teachers did not show significant difference in their ratings (*p* = 0.263). This meant that expert teachers seemed to be more inclined to rate the high-transparency sample material as of high transparency, while experienced and novice teachers failed to do so.

### Follow-Up Interview

Interview data further revealed how high- and low-transparency samples were approached and understood by teachers of different experience. The two sample materials were chosen from textbooks that the research participants did not use in their own teaching. This may explain why all of the interview informants confirmed that they did not remember reading or using these activities and that 14 out of the 15 interview informants particularly mentioned that the materials were not from the textbooks they were using. Interestingly, although teachers of different experience approached the low-transparency material in similar ways and most of them considered it difficult to understand and interpret, they approached the high-transparency material in quite different ways as is shown in the following typical responses:

As this is a lot different from our own textbook, I need to refrain from my habitual way of looking at textbooks … I should stand in the shoes of the compiler to see what they would like students to achieve with the whole bunch of activities as well as with each of them. (expert teacher)
I find the materials weird … The activities are arranged in a strange way. And in my class I don’t usually ask reading comprehension questions that way … I find the logic unclear between adjacent activities. (experienced teacher)
I can vaguely understand the teaching objectives of the activities, because they [the objectives] seem to be the same as those in the textbook I am using in my own teaching … I don’t understand how these activities should be done so as to achieve the objectives. I’m quite confused about that. (novice teacher)

As can be seen, both experienced and novice teachers met difficulties in understanding and interpreting the high-transparency material, though for different reasons: novice teachers might lack certain pedagogical knowledge that informed them of the inherent link between activity procedures and corresponding objectives, thus failing to identify how they could be compatible in the sample, while experienced teachers seemed to be tremendously influenced by the “weird” and “strange” look of the new material, especially when they compared their own teaching practice (e.g., how they actually asked reading comprehension questions) with the practice suggested in the new material. This can be corroborated to some extent by what an expert remarked in the interview:

It is not easy to see *through* the look of these “new” activities and grasp the learning design that underpins these activities … Once you’ve known how to identify the connection between objectives and activities, you’ll be able to sort out the structure of learning, or how your teaching can be structured in the classroom. (expert teacher)

As can be seen from this extract, teachers might need certain *knowledge* to be able to figure out the inherent pedagogical logic embedded in the activities of the textbook. Therefore, it is quite likely that it is lack of pedagogical *knowledge* that made the high-transparency material look less transparent than it actually was to novice teachers, and that it is the inflexible mind-set of processing teaching materials that prevented experienced teachers from utilizing such *knowledge* as well as the high transparency of the new material in understanding and interpreting it. Compared with experienced teachers, expert teachers demonstrated a much stronger awareness that existing schemata in their cognition might influence them as they processed new materials and consequently succeeded in removing the effects of stereotype that hampered the perception of transparency.

## Discussion and Conclusion

So far, how teachers of different experience approach and understand teaching materials of different transparency has been investigated with both quantitative and qualitative methods. Results and findings have verified that transparency of textbook activities affects the degree of easiness teachers perceive as they understand and interpret them for pedagogical purposes. This corroborates with previous research that examines the relationship between transparency of textbook activities and their accessibility for teachers in pedagogical processing (Xu, 2010, 2021). More importantly, the current study has revealed the impact of teachers’ experience on their perception of the transparency of textbook activities: discrepancy may exist between transparency in materials design and transparency in teachers’ perception. Two factors have been discovered that may hamper teachers’ perception of transparency: lack of pedagogical knowledge about the relationship between teaching objectives, steps, and assessment (as is the case of novice teachers in this study), and strong existing cognitive schemata developed from the stereotype of processing a more familar set of teaching materials (as is the case of experienced teacher).

The results and findings of the current study indicate that experience, which has been examined as the more important independent variable, does not necessarily foster stronger abilities of understanding and interpreting teaching materials, whether they are designed to be of high transparency or not. In other words, experience entails not only an increase in pedagogical knowledge that supports cognitive processing of textbook activities but also certain mental schemata, or plainly speaking, habitual thinking patterns, that have developed from past materials use and then influence future use.

The results and findings of the study may further indicate that the transparency of textbook activities should be treated not only as a feature of teaching materials but also, when perceived by teachers, as a component or representation of teachers’ pedagogical content knowledge (Shulman, 1987). As the perception of transparency in itself requires both knowledge of the language to be taught and knowledge about how it should be taught, transparency can, at least, be used as an auxiliary measure or manifestation of teachers’ pedagogical content knowledge in an authentic classroom setting.

The study reported in this article is only a pilot for a project in progress, so there are a few limitations to note. For instance, the participants came from economically better-off cities, making them less representable considering situations in less developed areas in China. Despite the limitations, the study still carries some implications. First, more research efforts are needed to further address the interface between textbook features in design and teachers’ perception of them in reality. That is to say, teaching materials should not be studied as detached from their use in context (Xu, 2010; Tomlinson, 2012). Second, some educational reforms such as the implementation of a new curriculum may often involve replacement of textbooks. The way teachers view and use new textbooks is quite likely to be influenced by their previous experiences of using earlier ones. This also needs to be taken into consideration as a potential source of influence or even hindrance as the reform is progressed. Last but not least, experience, usually operationalized by the number of years in service, should always be treated as a complex construct that may encompass various psychological traits.

## Data Availability Statement

The raw data supporting the conclusions of this article will be made available by the authors, without undue reservation.

## Ethics Statement

The studies involving human participants were reviewed and approved by Ethics Committee of National Research Centre for Foreign Language Education, Beijing Foreign Studies University. The participants provided their written informed consent to participate in this study.

## Author Contributions

HX is solely responsible for the contribution in this submission.

## Conflict of Interest

The author declares that the research was conducted in the absence of any commercial or financial relationships that could be construed as a potential conflict of interest.
